# *UHRF2* as a genetic correlate of hospitalization in sickle cell disease

**DOI:** 10.1111/bjh.70172

**Published:** 2025-10-05

**Authors:** Mina Cintho Ozahata, Monike Oliveira, Isabel Gomes, Cláudia Maximo, Alessandra Ferraz, André Rolim Belisário, Daiane Souza, Natalia A. Braga, Ester Sabino, Brian Custer, Shannon Kelly, Carla Luana Dinardo, the REDS‐III Brazil SCD Cohort study and the TOPMed consortium

**Affiliations:** ^1^ University of São Paulo São Paulo Brazil; ^2^ Faculdade de Medicina da Universidade de São Paulo São Paulo Brazil; ^3^ University of Mina Gerais Belo Horizonte Brazil; ^4^ Hemorio Foundation Rio de Janeiro Brazil; ^5^ Fundação HEMOPE Recife Pernambuco Brazil; ^6^ Centro de Tecidos Biológicos de Minas Gerais, Fundação Hemominas Belo Horizonte Brazil; ^7^ Vitalant Research Institute San Francisco California USA; ^8^ UCSF Department of Laboratory Medicine San Francisco California USA; ^9^ UCSF Benioff Children's Hospital Oakland Oakland California USA; ^10^ Fundação Pró‐Sangue São Paulo Brazil

**Keywords:** genetics, gwas, sickle cell disease

To the Editor,

Sickle cell disease (SCD), or sickle cell anemia, is the most prevalent genetic disease and represents a significant health burden worldwide. In 2021, an estimated 7.74 million people worldwide were living with SCD.^1^ In Brazil, the birth prevalence is 1 in 1000, resulting in about 3500 new cases annually. Around 45 000 people are living with SCD in Brazil, representing a significant public health challenge. Hydroxyurea (hydroxycarbamide) and chronic transfusion therapy (CTT) are the main disease‐modifying treatments.^2^, ^3^, ^4^, ^5^ Haematopoietic stem cell transplant^6^, ^7^ and gene therapy^8^, ^9^ may be curative but are not yet widely available.

SCD is characterized by haemolysis, chronic inflammation and vaso‐occlusive events, which are associated with multiorgan damage. Although most of the children will survive into adulthood, relative to children without SCD, childhood mortality is high.^1^ Up to 5% of children with SCD may die due to complications such as acute chest syndrome (ACS), sepsis, splenic sequestration, stroke and aplastic crisis.^10^, ^11^

Life expectancy in adulthood is also reduced. Adults with SCD often experience chronic pain and organ damage, with end‐stage organ failure being a potential cause of death. In Brazil, individuals with SCD died at a median age of 32.0 years, which is 37 years younger than the median age of death in the general population (69.0 years).^12^ In our large Brazilian cohort of individuals with SCD—part of the Recipient Epidemiology and Donor Evaluation Study‐III (REDS‐III) international programme—hospitalization was independently associated with an increased risk of death.^13^

The phenotypic expression of SCD varies widely. Modifiers of SCD severity have been identified, mainly the *β‐globin* genotype (such as HbSS, HbSC or others), the co‐inheritance of α‐thalassaemia trait and the amount of HbF production.^14^ However, the clinical manifestations of SCD vary even within the same *β‐globin* genotype group receiving identical disease‐modifying treatments, highlighting the potential role of other genetic factors underlying disease complications.

Although pain is commonly used as a proxy for SCD severity, it represents only part of the disease burden. Given the range of other clinical complications, hospitalization is common and associated with significant disease mortality. The rate of hospital readmissions, which is a quality‐of‐care indicator, is also significantly higher among patients with SCD in comparison with other patient groups.^15^ Disease‐modifying treatments, which reduce the morbimortality and the occurrence of the most important clinical complications of SCD, are known to prevent hospitalizations and to reduce the length of hospital stay in SCD.^16^, ^17^

Given the above, the frequency of hospitalizations in the SCD patient population can be considered an interesting marker of disease severity. Our objective was to identify the genetic markers associated with SCD disease severity using hospitalizations as a marker of severity. To this end, a Genome‐wide association study (GWAS) was conducted in a large, well‐ Brazilian cohort.

Participants were drawn from the REDS‐III Brazil SCD cohort, part of the National Heart, Lung, and Blood Institute (NHLBI)'s REDS‐III. A total of 2793 individuals with SCD were enrolled. Clinical, demographic and laboratory data were collected following as previously described.^18^

Homozygous SS and HbSβ0‐thalassaemia individuals were selected for the analysis and all‐cause frequent hospitalization as a marker of SCD disease severity based on the diverse phenotypes leading to hospitalization. Data on hospitalizations were collected during study enrolment and two consecutive follow‐up visits (2013–2015). Hospitalization was defined as ≥24 h of hospital stay. For each hospitalization, a maximum of three diagnoses were registered. We defined a composite dichotomous predictor of participants with ≥3 inpatient hospitalizations in the study period compared to participants with <3.

Whole‐genome sequencing, performed by the NHLBI Trans‐Omics for Precision Medicine (TOPMed) programme, has been described elsewhere.^19^ Association analysis, as well as Q–Q and Manhattan plots, was performed using ENCORE (https://encore.sph.umich.edu). The SAIGE (Scalable and Accurate Implementation of GEneralized mixed model)^20^ logistic mixed model was used. Adjustments were made for hydroxyurea (hydroxycarbamide) treatment and the first 10 principal components.

A genome‐wide *p*‐value threshold of 5 × 10^−8^ was applied. Only single nucleotide variants (SNVs) with a Minor allele frequency greater than 0.1% were included. LocusZoom^21^ was used to generate LocusZoom plots.

To investigate whether a secondary signal existed beyond the lead SNV, a stepwise conditional analysis using GCTA‐COJO^22^ was conducted. To identify putative causal variants within the UHRF2 gene region, variant‐level statistical fine‐mapping using Sum of single effects (SuSIE) regression (SuSiE‐R)^23^ with summary statistics was performed. SNVs that reached genome‐wide significance were tested using the chi‐square test for associations with mortality during the study period.

Of the entire cohort, a subset of 1931 participants with HbSS or HbSβ0 genotypes had available data on whole‐genome sequencing, hospitalization history, age, sex and hydroxyurea (hydroxycarbamide) treatment and were included in this analysis. Of these, 387 (20%) had ≥3 hospitalizations in the ~3‐year follow‐up period who were compared to 1544 other cohort members. The majority of the participants (44.3%) did not have any hospitalizations in the 3‐year period. One hundred and seventy‐one participants had 5 or more hospitalizations.

There were no significant differences in the sex and age distributions of participants with ≥3 hospitalizations compared to participants with <3 hospitalizations (54% vs. 49.7% female and 18.8 years vs. 20.6 years respectively). There were also no significant differences in haemoglobin levels, lactate dehydrogenase, platelet counts or the prevalence of stroke, leg ulcers and priapism between the two groups. However, participants with ≥3 hospitalizations were more likely to be treated with hydroxyurea (hydroxycarbamide) and received more transfusions in addition to having a higher prevalence of complications such as ACS, hospitalization by vaso‐occlusive pain, avascular necrosis and kidney disease, suggesting a more severe phenotype of these individuals (demographic and clinical history are summarized in Table 1).

**TABLE 1:** Characteristics of participants included in the REDS‐III Brazil sickle cell disease cohort study, stratified by the occurrence of ≥3 hospitalizations within 1 year (*N* = 1931).

Variables^a^	<3 hospitalizations per year (*n* = 1544) mean (SD) for continuous measures or *N* (%) for categorical measures	≥3 hospitalizations per year (*n* = 387) mean (SD) for continuous measures or *N* (%) for categorical measures	*p*‐value
Age (years/mean (SD))^&^	20.6 (13.5)	18.8 (12.2)	0.06
Sex (Female) (*N* (%))	833 (54)	193 (49.7)	0.1
Treatment with hydroxyurea (hydroxycarbamide) (*N* (%))^#^	821 (53.2)	259 (66.7)	<0.001
Transfusions in study period (*N* (%))^&^	3.6 (8)	4.5 (7.6)	<0.001
Stroke (*N* (%))^#^	181 (11.7)	32 (8.2)	0.06
Lifetime history of acute chest syndrome (*N* (%))^#^	1032 (66.8)	340 (87.6)	<0.001
Lifetime history of hospitalization by pain crisis (*N* (%))^#^	455 (29.5)	359 (92.5)	<0.001
Avascular necrosis (*N* (%)) ^#^	146 (9.5)	62 (16)	<0.001
Haemoglobin (g/dL/Mean (SD))^&^	8.4 (1.3)	8.4 (1.2)	0.3
Lactate dehydrogenase (U/L/Mean (SD))^&^	662 (422.2)	618.5 (370.9)	0.06
Platelets (mm^3^/Mean (SD))^&^	418724.3 (130959.5)	426529.0 (13278.7)	0.4
Kidney disease (*N* (%)) ^#^	77 (0.5)	33 (0.9)	0.01
Leg ulcers (*N* (%)) ^#^	175 (11.3)	50 (12.9)	0.4
Priapism (*N* (%)) ^#^	135 (8.7)	34 (8.8)	0.7

*Note*: Variables marked with # were evaluated using chi‐squared test and variables marked with & were evaluated with Mann–Whitney test.

^a^ In the first column of the table, in addition to the variable, we included the unit of measurement in parentheses as well as whether the values are presented as mean (SD) or *N* (%). Study participants were between 1 and 75 years of age.

A total of 2955 hospitalizations were recorded, with up to three diagnoses reported for each hospitalization (Figure 1). Most hospitalizations were related to SCD pain, and the second most frequent diagnosis was ACS (Figure 2).

GWAS was performed comparing the groups with ≥3 hospitalizations to those with fewer than 3, adjusting for hydroxyurea (hydroxycarbamide) treatment. Figure 3 provides a summary of the GWAS results. The Q–Q plot (Figure 3) showed no evidence of bias in *p*‐values and the *λ*_GC_ (=1.097) indicated no genomic inflation or additional confounding factors. A total of 20 SNVs (Table 2 and Figure S1) reached genome‐wide significance and are located in chromosome 9 near or in gene *ubiquitin‐like with PHD and ring finger domains 2* (*UHRF2*) (Figure S1). The SNVs also showed significant associations with mortality during the study period (Table S1), underscoring the potential relevance of this locus to disease severity (Figures 2 and 3).

**FIGURE 1:**
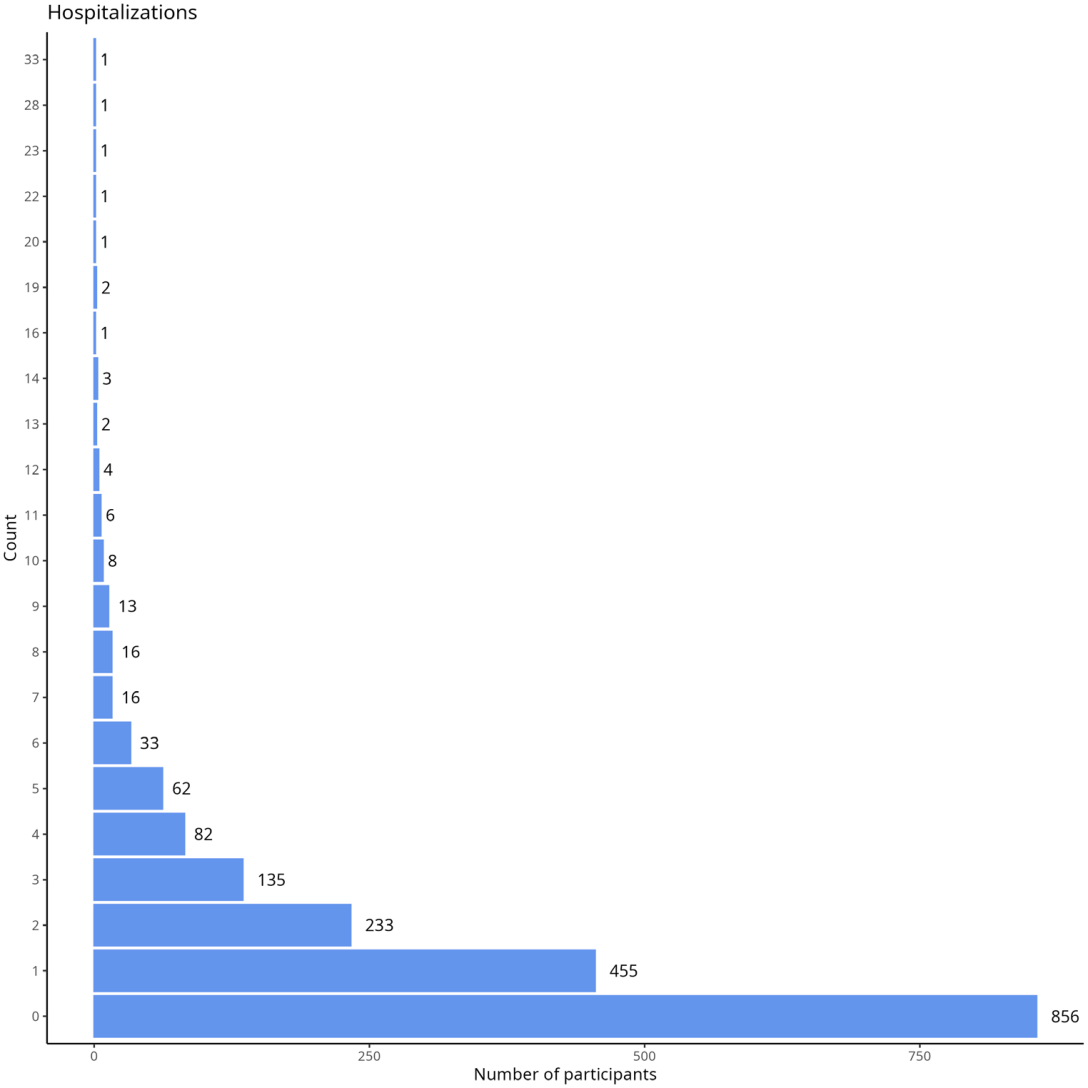
Distribution of the number of hospitalizations per patient.

**FIGURE 2:**
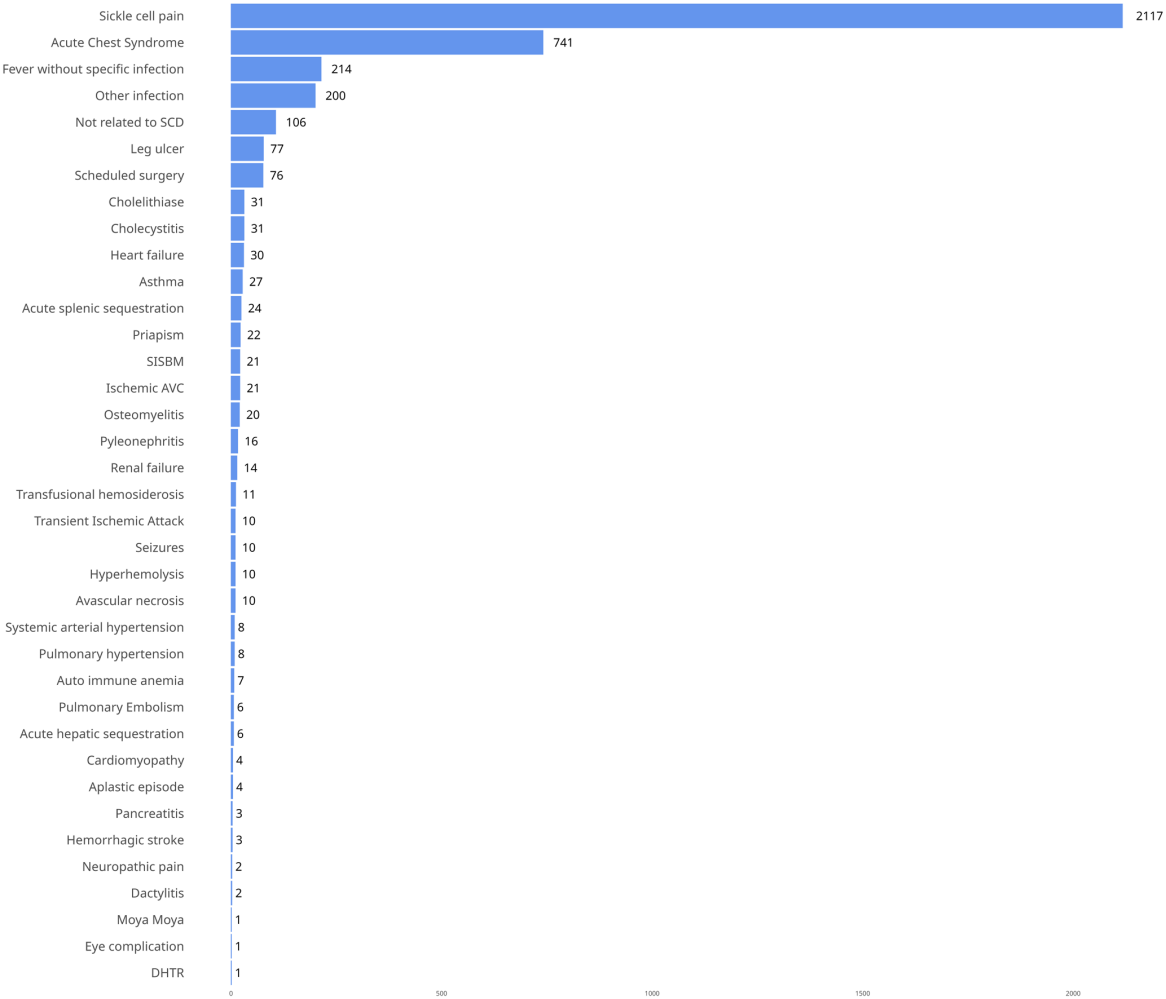
Most common diagnoses associated with hospitalizations. A total of 2955 hospitalizations were recorded in the 1931 participants, with up to three diagnoses documented for each hospitalization. DHTR, delayed haemolytic transfusion reaction; SISBM, serious infection, sepsis, bacteraemia or meningitis.

**FIGURE 3:**
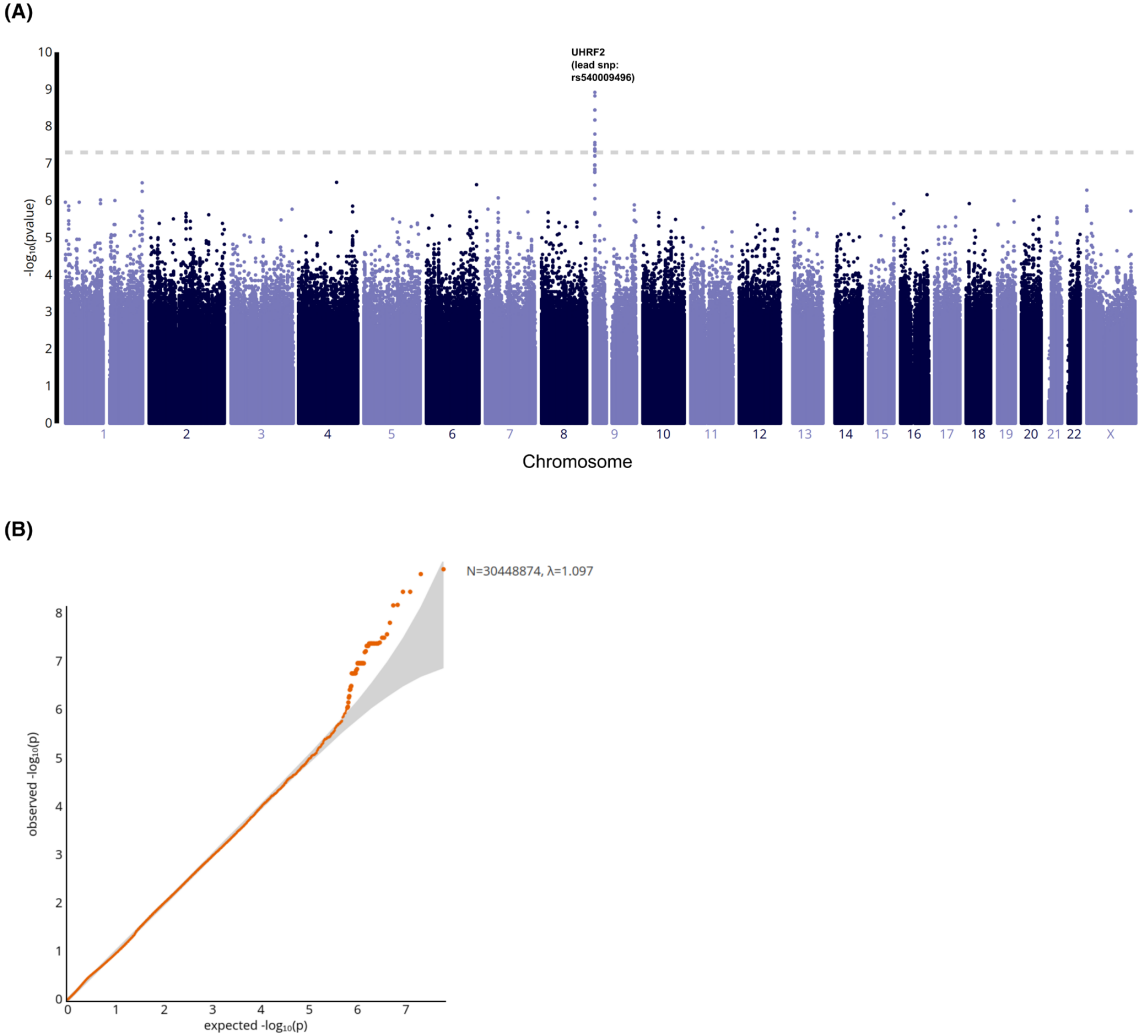
(A) A Manhattan plot displays the genome‐wide −log_10_ *p*‐values plotted against the positions of SNVs on each chromosome, showing the association between SNVs and the number of hospitalizations. The grey horizontal line represents the threshold for genome‐wide significance at 5 × 10^−8^. (B) Q–Q plot of the observed (*y* axis) versus expected (*x* axis) –log (*p*‐values). The genomic inflation factor (*λ*) is 1.097, indicating minimal inflation and suggesting that population stratification or other confounding factors are well‐controlled. Sample size: *N* = 30 448 874 SNVs. The tail deviation observed in the Q–Q plot indicates the presence of several genome‐wide significant associations.

From the conditional analysis, no additional independent significant SNVs were identified after conditioning on the lead SNV. SuSiE‐R identified two 95% credible sets, with a posterior inclusion probability of 1, each containing a single SNV: rs201474308 and rs1330381, respectively (Figure S2), suggesting the presence of at least two independent signals.

*UHRF* proteins take part in important biological processes, including maintenance of DNA methylation, cell cycle regulation and DNA damage repair.^24^, ^25^ There is a notable body of evidence linking the *UHRF* family of proteins with cellular regulation, including development, gametogenesis and carcinogenesis.^26^ *UHRF2* has been extensively associated with gene expression regulation,^27^ including haematopoiesis.^28^ In a mouse model, a *UHRF2* knockout genotype showed an impaired ability of haematopoietic stem/progenitor cells (HSPCs) to reconstitute haematopoiesis (Table 2).

**TABLE 2:** Top SNVs in chromosome 9 associated with number dichotomous hospitalizations (<3 compared to ≥3) with genome‐wide significance (*p* < 10^−8^).

Position	SNV id	BETA	*p*‐value for the association with the number of hospitalizations	Risk alleles	Minor allele frequency (MAF)	*p*‐value for the association with mortality during the study period
6 478 035	rs540009496	3.15	1.21e‐09	C	0.0078	1e‐10
6 500 956	rs115251053	3.11	1.51e‐09	A	0.0078	3e‐11
6 446 477	rs7030161	2.99	3.56e‐09	T	0.008	1e‐10
6 494 612	rs146803792	2.99	3.56e‐09	G	0.008	1e‐10
6 502 294	rs147106460	2.91	6.65e‐09	G	0.008	4e‐10
6 483 804	rs192217552	2.91	6.69e‐09	G	0.008	5e‐10
6 479 871	rs114749020	2.79	1.56e‐08	G	0.009	1e‐10
6 429 155	rs539055817	2.51	2.69e‐08	A	0.01	5e‐9
6 373 683	rs201474308	2.07	3.13e‐08	TA	0.0145	1e‐10
6 371 594	rs183470719	2.59	3.14e‐08	G	0.01	3e‐9
6 309 855	rs115678288	2.10	3.97e‐08	C	0.014	4e‐11
6 347 334	rs570833262	2.16	4.13e‐08	A	0.013	6e‐11
6 349 335	rs1599369	2.16	4.13e‐08	T	0.013	6e‐11
6 371 925	rs115081564	2.16	4.13e‐08	G	0.013	6e‐11
6 384 592	rs140602739	2.16	4.13e‐08	G	0.013	6e‐11
6 394 725	rs143965715	2.16	4.13e‐08	G	0.013	6e‐11
6 397 055	rs143101370	2.16	4.13e‐08	G	0.013	6e‐11
6 402 347	rs141243822	2.16	4.13e‐08	T	0.013	6e‐11
6 382 252	rs369459407	2.45	4.63e‐08	A	0.010	1e‐9
6 413 029	rs569463884	2.45	4.63e‐08	G	0.010	1e‐9

The role of *UHRF* proteins in maintaining DNA methylation is important to highlight. Dysregulation of the methylation process caused by either genetic mutations or environmental stimuli is potentially associated with multiple diseases and accelerates ageing.^29^ A discrepancy between epigenetic age and chronological age, that is called ‘epigenetic age acceleration’, underlies higher mortality risk and age‐related conditions in humans.^30^ Additionally, DNA methylation is associated with HbF levels and the response to hydroxyurea (hydroxycarbamide), involving the methylation of γ‐globin genes by DNA methyltransferases (*DNMT3A, DNMT3B, DNMT1* and *MBD* family).^31^ Both *UHRF1* and *UHRF2* interact with *DNMT1, DNMT3a, DNMT3b* and *G9a*.^32^ On average, individuals with SCD have at least a 20‐year reduction in survival compared to the overall population, with a life expectancy of 52.6 years.^33^

*UHRF2* has been identified as a potential biomarker for elevated tricuspid regurgitation velocity in SCD.^34^ The observed association in Desai et al. suggests a possible role for *UHRF2* in the pathogenesis of pulmonary hypertension, one of the more severe organ complications presented by patients with SCD. Alternatively, *UHRF2* may take part in pulmonary vaso‐occlusions, which underlie the occurrence of pulmonary hypertension. Cardiopulmonary complications are a major cause of death in adults with SCD, and elevated estimated pulmonary artery systolic pressure has been associated with increased mortality.^35^

Our identified association between the UHRF protein family and SCD severity suggests epigenetics as a factor underlying SCD burden, potential organ damage and longevity. Identifying the genetic background of SCD severity is important to the identification and prognosis of individuals who may be candidates for transformative therapies such as stem cell transplantation or gene therapy. If confirmed in other large cohorts, our finding of variations in *UHRF2* as a potential marker of disease severity, associated epigenetic modulation and pulmonary hypertension may help identify these higher‐risk individuals.

We explored genetic factors associated with frequent hospitalizations in individuals with SCD, using this outcome as a proxy for disease severity. The identified association between *UHRF2* and SCD severity helps our understanding of the genetics underlying an unfavourable disease course in SCD. Further studies are needed to investigate mechanisms and pathways through which the *UHRF2* influences SCD severity.

## AUTHOR CONTRIBUTIONS

Mina Cintho Ozahata cleaned, analysed and interpreted the data, and drafted the manuscript. Isabel cleaned and analysed the data, and approved the final manuscript. Monike Oliveira, Cláudia Maximo, Alessandra Ferraz, André Rolim Belisário and Daiane Souza collected the data, contributed to data interpretation, and reviewed and edited the manuscript. Shannon Kelly, Ester C. Sabino and Brian Custer supervised the protocol design, and reviewed, edited and approved the manuscript. Carla L. Dinardo conceptualized the study, and supervised, reviewed, edited and approved the manuscript.

## FUNDING INFORMATION

Molecular data for the Trans‐Omics in Precision Medicine (TOPMed) programme was supported by the National Heart, Lung, and Blood Institute (NHLBI). WGS for ‘NHLBI TOPMed: REDSIII’ (phs001468) was performed at the University of Washington, Seattle, WA (HHSN268201500015C).

REDS‐III was supported by the National Institutes of Health, the National Heart, Lung, and Blood Institute under Grants HHSN2682011‐00001I, ‐00002I, ‐00003I, ‐00004I, ‐00005I, ‐00006I, ‐00007I, ‐00008I and ‐00009I.

## CONFLICT OF INTEREST STATEMENT

The authors have no conflict of interest with regard to the publication of this manuscript.

## ETHICS STATEMENT

This research was approved by all relevant ethical committees/IRBs in both the US and Brazil, including the Brazilian National Committee of Ethics in Research, local ethical committees at each participating site as well as the IRB of record for Vitalant Research Institute.

## PATIENT CONSENT STATEMENT

All participants (or guardians of participants <18 years) signed informed consent to participate in the REDS‐III Brazil SCD cohort and allowed their DNA and data to be used in GWAS.

## Supporting information


Figure S1.

Figure S2.

Table S1.

## Data Availability

Data collected in the Recipient Epidemiology and Donor Evaluation Study (REDS‐III) is available through the American National Institute of Health database of Genotypes and Phenotypes (dbGaP) upon request.

## References

[1] Thomson AM , McHugh TA , Oron AP , Teply C , Lonberg N , Vilchis Tella V , et al. Global, regional, and national prevalence and mortality burden of sickle cell disease, 2000–2021: a systematic analysis from the global burden of disease study 2021. Lancet Haematol. 2023;10(8):e585–e599. 37331373 10.1016/S2352-3026(23)00118-7 PMC10390339

[2] Nevitt SJ , Jones AP , Howard J . Hydroxyurea (hydroxycarbamide) for sickle cell disease. Cochrane Database Syst Rev. 2017;2017(4):CD002202. 10.1002/14651858.CD002202.pub2 PMC6478259 28426137

[3] Rankine‐Mullings AE , Nevitt SJ . Hydroxyurea (hydroxycarbamide) for sickle cell disease. Cochrane Database Syst Rev. 2022;2022(10):CD002202. 10.1002/14651858.CD002202.pub3 PMC9435593 36047926

[4] McGann PT , Ware RE . Hydroxyurea therapy for sickle cell anemia. Expert Opin Drug Saf. 2015;14(11):1749–1758. 26366626 10.1517/14740338.2015.1088827 PMC5868345

[5] Quinn CT , Ware RE . The modern use of hydroxyurea for children with sickle cell anemia. Haematology. 2025;110(5):1061–1073. https://haematologica.org/article/view/11891 10.3324/haematol.2023.284633 PMC12050929 39781621

[6] Lucarelli G , Gaziev J , Isgrò A , Sodani P , Paciaroni K , Alfieri C , et al. Allogeneic cellular gene therapy in hemoglobinopathies—evaluation of hematopoietic SCT in sickle cell anemia. Bone Marrow Transplant. 2012;47(2):227–230. 21499319 10.1038/bmt.2011.79

[7] Tanhehco YC , Bhatia M . Hematopoietic stem cell transplantation and cellular therapy in sickle cell disease: where are we now? Curr Opin Hematol. 2019;26(6):448–452. 31483336 10.1097/MOH.0000000000000541

[8] Germino‐Watnick P , Hinds M , Le A , Chu R , Liu X , Uchida N . Hematopoietic stem cell gene‐addition/editing therapy in sickle cell disease. Cells. 2022;11(11):1843. 35681538 10.3390/cells11111843 PMC9180595

[9] Anurogo D , Budi YP , Ngo TMH , Huang YH , Pawitan JA . Cell and gene therapy for anemia: hematopoietic stem cells and gene editing. IJMS. 2021;22(12):6275. 34200975 10.3390/ijms22126275 PMC8230702

[10] Quinn CT , Rogers ZR , McCavit TL , Buchanan GR . Improved survival of children and adolescents with sickle cell disease. Blood. 2010;115(17):3447–3452. 20194891 10.1182/blood-2009-07-233700 PMC2867259

[11] Neonato MG , Guilloud‐Bataille M , Beauvais P , Bégué P , Belloy M , Benkerrou M , et al. Acute clinical events in 299 homozygous sickle cell patients living in France. Eur J Haematol. 2000;65(3):155–164. 11007050 10.1034/j.1600-0609.2000.90210.x

[12] Cançado RD , Costa FF , Lobo C , Migliavaca CB , Falavigna M , Souza Filho HCR , et al. Estimated mortality rates of individuals with sickle cell disease in Brazil: real‐world evidence. Blood Adv. 2023;7(15):3783–3792. 37104056 10.1182/bloodadvances.2022008938 PMC10393747

[13] Caselli PFB , Di Lorenzo OC , Gomes I , Salomon T , Sabino EC , Capuani L , et al. Mortality from sickle cell disease in Brazil. PLoS Glob Public Health. 2025;5(7):e0002066. 40705788 10.1371/journal.pgph.0002066 PMC12289050

[14] Rees DC , Brousse VAM , Brewin JN . Determinants of severity in sickle cell disease. Blood Rev. 2022;56:100983. 35750558 10.1016/j.blre.2022.100983

[15] Goel R , Yang P , Zhu X , Patel EU , Crowe EP , Rai H , et al. Hospital readmissions among people with sickle cell disease. JAMA Netw Open 2025;8(6):e2517974. 40526379 10.1001/jamanetworkopen.2025.17974 PMC12175023

[16] Steinberg MH , McCarthy WF , Castro O , Ballas SK , Armstrong FD , Smith W , et al. The risks and benefits of long‐term use of hydroxyurea in sickle cell anemia: a 17.5 year follow‐up. American J Hematol. 2010;85(6):403–408. 10.1002/ajh.21699 PMC2879711 20513116

[17] Payne J , Aban I , Hilliard LM , Madison J , Bemrich‐Stolz C , Howard TH , et al. Impact of early analgesia on hospitalization outcomes for sickle cell pain crisis. Pediatr Blood Cancer. 2018;65(12):e27420. 30151977 10.1002/pbc.27420 PMC6192851

[18] ABF C‐P , Kelly S , Miranda Teixeira C , Sabino EC , Alencar CS , Capuani L , et al. Clinical and genetic ancestry profile of a large multi‐centre sickle cell disease cohort in Brazil. Br J Haematol. 2018;182(6):895–908. 30027669 10.1111/bjh.15462 PMC8019534

[19] Taliun D , Harris DN , Kessler MD , Carlson J , Szpiech ZA , Torres R , et al. Sequencing of 53,831 diverse genomes from the NHLBI TOPMed Program. Nature. 2021;590(7845):290–299. 33568819 10.1038/s41586-021-03205-y PMC7875770

[20] Zhou W , Nielsen JB , Fritsche LG , Dey R , Gabrielsen ME , Wolford BN , et al. Efficiently controlling for case‐control imbalance and sample relatedness in large‐scale genetic association studies. Nat Genet. 2018;50(9):1335–1341. 30104761 10.1038/s41588-018-0184-y PMC6119127

[21] Boughton AP , Welch RP , Flickinger M , VandeHaar P , Taliun D , Abecasis GR , et al. LocusZoom.js: interactive and embeddable visualization of genetic association study results. Marschall T, editor. Bioinformatics. 2021;37(18):3017–3018. 33734315 10.1093/bioinformatics/btab186 PMC8479674

[22] Svishcheva GR , Belonogova NM , Kirichenko AV , Tsepilov YA , Axenovich TI . A New Method for Conditional Gene‐Based Analysis Effectively Accounts for the Regional Polygenic Background. Gen. 2024;15(9):1174. 10.3390/genes15091174 PMC11431718 39336765

[23] Zou Y , Carbonetto P , Wang G , Stephens M . Fine‐mapping from summary data with the “Sum of Single Effects” model. Epstein MP, editor. PLoS Genet. 2022;18(7):e1010299. 35853082 10.1371/journal.pgen.1010299 PMC9337707

[24] Zeng S , Wang Y , Zhang T , Bai L , Wang Y , Duan C . E3 ligase UHRF2 stabilizes the acetyltransferase TIP60 and regulates H3K9ac and H3K14ac via RING finger domain. Protein Cell. 2017;8(3):202–218. 27743347 10.1007/s13238-016-0324-z PMC5326618

[25] Vaughan RM , Dickson BM , Cornett EM , Harrison JS , Kuhlman B , Rothbart SB . Comparative biochemical analysis of UHRF proteins reveals molecular mechanisms that uncouple UHRF2 from DNA methylation maintenance. Nucleic Acids Res. 2018;46(9):4405–4416. 29506131 10.1093/nar/gky151 PMC5961305

[26] Demirdizen E , Al‐Ali R , Narayanan A , Sun X , Varga JP , Steffl B , et al. TRIM67 drives tumorigenesis in oligodendrogliomas through Rho GTPase‐dependent membrane blebbing. Neuro‐Oncology. 2023;25(6):1031–1043. 36215168 10.1093/neuonc/noac233 PMC10237422

[27] Liu Y , Zhang B , Kuang H , Korakavi G , Lu LY , Yu X . Zinc Finger Protein 618 Regulates the Function of UHRF2 (Ubiquitin‐like with PHD and Ring Finger Domains 2) as a Specific 5‐Hydroxymethylcytosine Reader. J Biol Chem. 2016;291(26):13679–13688. 27129234 10.1074/jbc.M116.717314 PMC4919451

[28] Sano T , Ueda K , Minakawa K , Mori T , Hashimoto Y , Koseki H , et al. Impaired Repopulating Ability of Uhrf2−/− Hematopoietic Progenitor Cells in Mice. Gen. 2023;14(8):1531. 10.3390/genes14081531 PMC10454722 37628583

[29] Liu R , Zhao E , Yu H , Yuan C , Abbas MN , Cui H . Methylation across the central dogma in health and diseases: new therapeutic strategies. Sig Transduct Target Ther. 2023;8(1):310. 10.1038/s41392-023-01528-y PMC10449936 37620312

[30] Horvath S , Haghani A , Zoller JA , Lu AT , Ernst J , Pellegrini M , et al. Pan‐primate studies of age and sex. Geroscience. 2023;45(6):3187–3209. 37493860 10.1007/s11357-023-00878-3 PMC10643767

[31] Lewis J , Guilcher GMT , Greenway SC . Reviewing the impact of hydroxyurea on DNA methylation and its potential clinical implications in sickle cell disease. Eur J Haematol. 2024;113(3):264–272. 38831675 10.1111/ejh.14247

[32] Zhang J , Gao Q , Li P , Liu X , Jia Y , Wu W , et al. S phase‐dependent interaction with DNMT1 dictates the role of UHRF1 but not UHRF2 in DNA methylation maintenance. Cell Res. 2011;21(12):1723–1739. 22064703 10.1038/cr.2011.176 PMC3357991

[33] Jiao B , Johnson KM , Ramsey SD , Bender MA , Devine B , Basu A . Long‐term survival with sickle cell disease: a nationwide cohort study of Medicare and Medicaid beneficiaries. Blood Adv. 2023;7(13):3276–3283. 36929166 10.1182/bloodadvances.2022009202 PMC10336259

[34] Desai AA , Zhou T , Ahmad H , Zhang W , Mu W , Trevino S , et al. A novel molecular signature for elevated tricuspid regurgitation velocity in sickle cell disease. Am J Respir Crit Care Med. 2012;186(4):359–368. 22679008 10.1164/rccm.201201-0057OC PMC3443809

[35] Gladwin MT , Sachdev V , Jison ML , Shizukuda Y , Plehn JF , Minter K , et al. Pulmonary hypertension as a risk factor for death in patients with sickle cell disease. N Engl J Med. 2004;350(9):886–895. 14985486 10.1056/NEJMoa035477

