# Supplementary figures and images for: UHRF2 as a genetic correlate of hospitalization in sickle cell disease
Source: Br J Haematol. 2025 Oct 5;207(6):2622–8. doi: 10.1111/bjh.70172 (PMC12710194; doi:10.1111/bjh.70172)

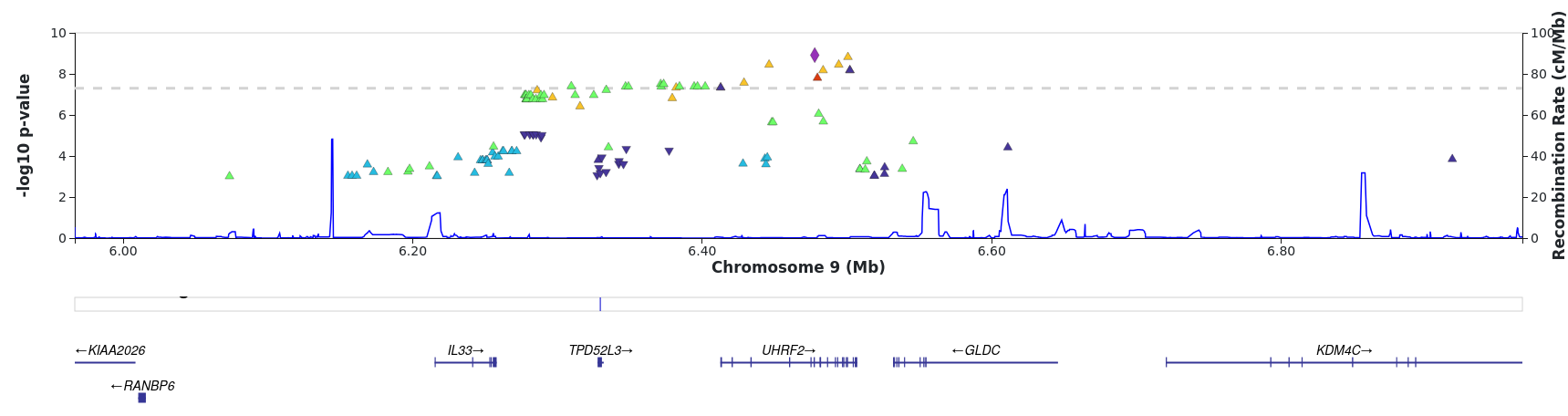

Supplement: Supplementary file 1 — Figure S1. [file BJH-207-2622-s003.png]

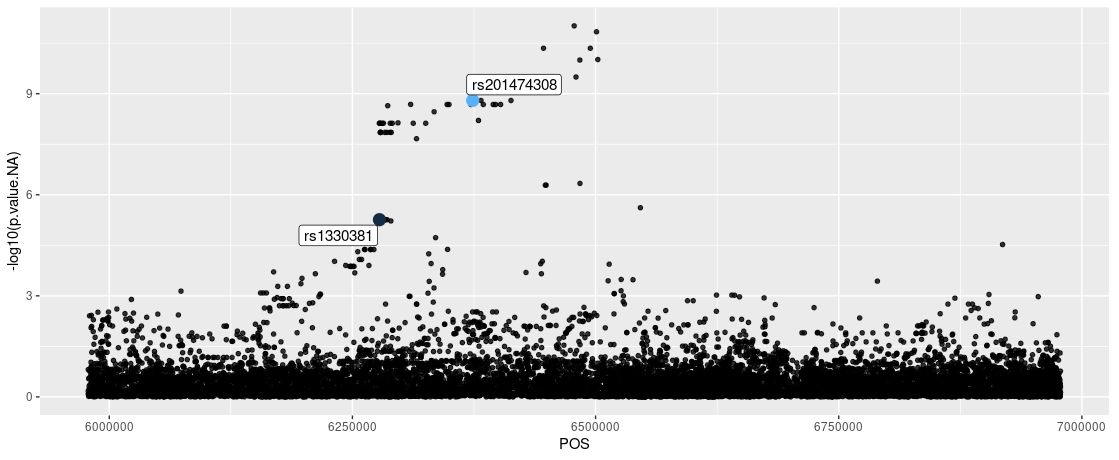

Supplement: Supplementary file 2 — Figure S2. [file BJH-207-2622-s001.png]
