# Supplementary material for: UHRF2 as a genetic correlate of hospitalization in sickle cell disease
Source: Br J Haematol. 2025 Oct 5;207(6):2622–8. doi: 10.1111/bjh.70172 (PMC12710194; doi:10.1111/bjh.70172)
Supplement: Supplementary file 3 — Table S1. [file BJH-207-2622-s002.docx]

**Supplementary Table 1:** Top SNVs in chromosome 9 associated with number dichotomous hospitalizations (<3 compared to ≥3) with genome-wide significance (P < 10^-8^).

| **Position** | **SNP id** | **BETA** | **P-value for the association with the number of hospitalizations** | **Risk alleles** | **Minor allele frequency (MAF)** | **P-value for the association with mortality during the study period (chi-squared test)** |
| --- | --- | --- | --- | --- | --- | --- |
| 6478035 | rs540009496 | 3.15 | 1.21e-09 | C | 0,0078 | 1e-10 |
| 6500956 | rs115251053 | 3.11 | 1.51e-09 | A | 0,0078 | 3e-11 |
| 6446477 | rs7030161 | 2.99 | 3.56e-09 | T | 0,008 | 1e-10 |
| 6494612 | rs146803792 | 2.99 | 3.56e-09 | G | 0,008 | 1e-10 |
| 6502294 | rs147106460 | 2.91 | 6.65e-09 | G | 0,008 | 4e-10 |
| 6483804 | rs192217552 | 2.91 | 6.69e-09 | G | 0,008 | 5e-10 |
| 6479871 | rs114749020 | 2.79 | 1.56e-08 | G | 0,009 | 1e-10 |
| 6429155 | rs539055817 | 2.51 | 2.69e-08 | A | 0,01 | 5e-9 |
| 6373683 | rs201474308 | 2.07 | 3.13e-08 | TA | 0,0145 | 1e-10 |
| 6371594 | rs183470719 | 2.59 | 3.14e-08 | G | 0,01 | 3e-9 |
| 6309855 | rs115678288 | 2.10 | 3.97e-08 | C | 0,014 | 4e-11 |
| 6347334 | rs570833262 | 2.16 | 4.13e-08 | A | 0,013 | 6e-11 |
| 6349335 | rs1599369 | 2.16 | 4.13e-08 | T | 0,013 | 6e-11 |
| 6371925 | rs115081564 | 2.16 | 4.13e-08 | G | 0,013 | 6e-11 |
| 6384592 | rs140602739 | 2.16 | 4.13e-08 | G | 0,013 | 6e-11 |
| 6394725 | rs143965715 | 2.16 | 4.13e-08 | G | 0,013 | 6e-11 |
| 6397055 | rs143101370 | 2.16 | 4.13e-08 | G | 0,013 | 6e-11 |
| 6402347 | rs141243822 | 2.16 | 4.13e-08 | T | 0,013 | 6e-11 |
| 6382252 | rs369459407 | 2.45 | 4.63e-08 | A | 0,010 | 1e-9 |
| 6413029 | rs569463884 | 2.45 | 4.63e-08 | G | 0,010 | 1e-9 |

**Supplementary Figure 1.**


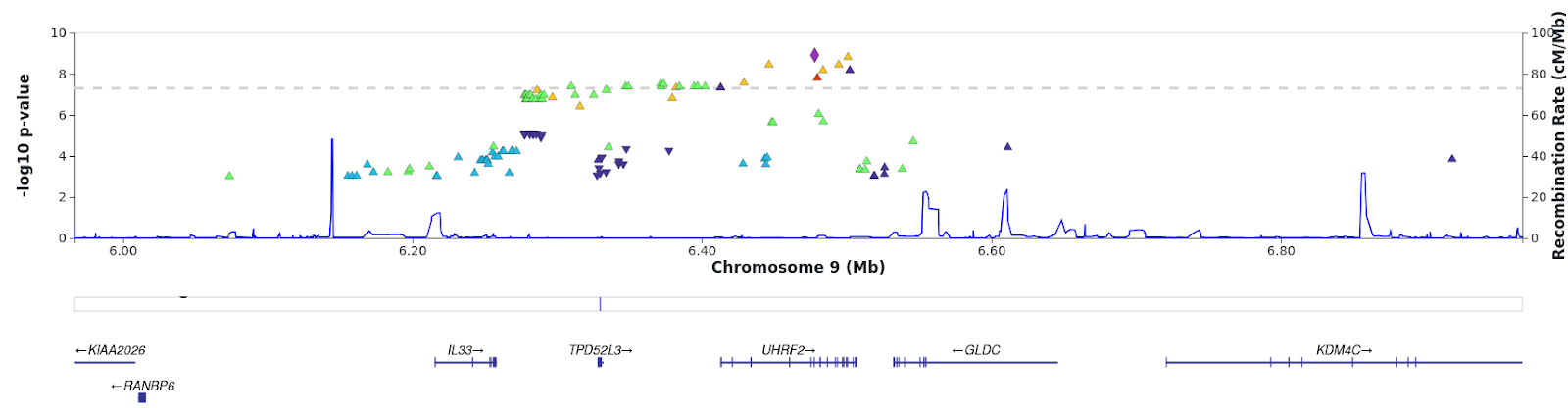


A regional LocusZoom plot of chromosome 9 is presented, where the y-axis represents the negative logarithm of the p-values for the association between SNPs and the number of hospitalizations. Each SNP is shown as a dot. The x-axis represents the relative positions of these SNPs within the chromosome region, with the values reflecting the recombination rate in centimorgans (cM) per megabase (Mb). The colors of the dots indicate the level of linkage disequilibrium (LD) with rs115251053, which is marked by a purple diamond. The LD is measured using

**Supplementary Figure 2.**


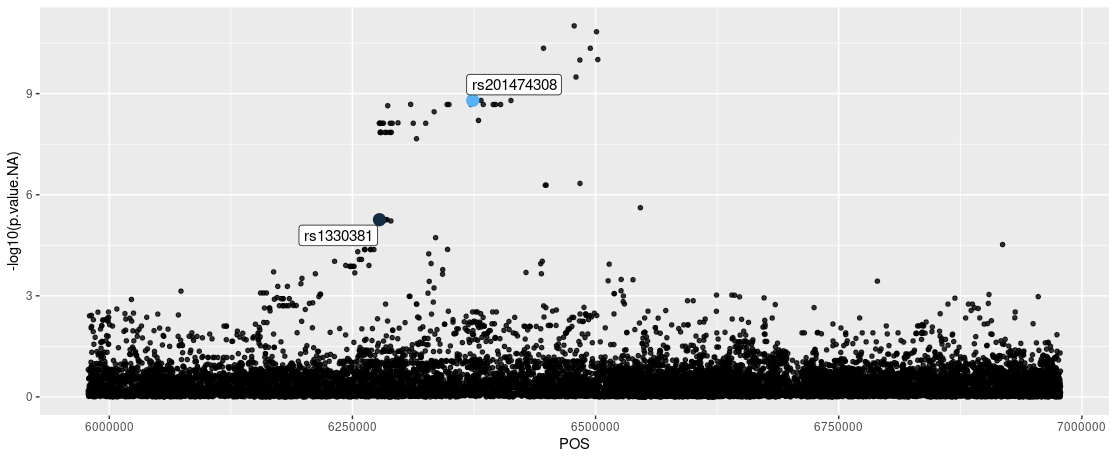


A regional plot of chromosome 9 with the two SNVs in the credible sets (rs201474308 and rs1330381) resulting from the statistical fine-mapping analysis using SuSiE regression. The plot identifies two distinct credible sets within the UHRF2 gene region, each representing a group of variants with high posterior probabilities of being causal.
